# Supplementary material for: Newly Arrived Migrant Women’s Experience of Maternity Health Information: A Face-to-Face Questionnaire Study in Norway
Source: Int J Environ Res Public Health. 2021 Jul 15;18(14):7523. doi: 10.3390/ijerph18147523 (PMC8307311; doi:10.3390/ijerph18147523)
Supplement: Supplementary file 1 [file ijerph-18-07523-s001.zip › File S1.pdf]

MIPREG QUESTIONNAIRE

1. GENERAL

1.1 What country were you born in?

1.2. What country was the father of your child born in?

1.3. How long have you lived in Norway?

1.4. How old are you?

1.5. What language do you use most often at home?

1.6. How good is your Norwegian?

Oral –	Fluent	Good	Some difficulty	Not at all
Reading –	Fluent	Good	Some difficulty	Not at all
Writing –	Fluent	Good	Some difficulty	Not at all
Comprehension –	Fluent	Good	Some difficulty	Not at all

1.7. What is your postal code?

1.8. What is your marital status?

- Single
- Married/cohabiting
- Divorced
- Widow

1.9. Who do you live with?

- ☐ Partner
- ☐ Your family (your mother/father, your brother/sister)
- ☐ In-laws (parent in-law, your partner's brother/sister)
- ☐ Friends/colleagues
- ☐ Children (in addition to your newborn child)
- ☐ None, I live alone

1.10 a) Do you have anyone you trust with whom you can speak in confidence?

Yes

No

1.10 b) If the answer is YES, who is this person?

- Partner
- Your family (your mother/father, your brother/sister)
- In-laws (parent in-law, your partner's brother/sister)
- Friends/colleagues

1.11 What is the highest level of education you completed?

- I have no schooling
- Begun, but not completed compulsory education
- Primary/lower secondary school (first 7 to 10 years of schooling)
- High school/upper secondary (the next 1-3 years of education)
- Tertiary/university, short (up to 4 years)
- Tertiary/university, long (4 years or more)

1.12 Have you had paid work since coming to Norway?

- Yes
- No

1.13 In the course of the last 12 months, have you or your family had difficulties making ends meet and paying monthly expenses (food, transport, housing etc.)?

- Yes, often
- Yes, occasionally
- No, never
- Do not know/prefer not to answer

2. YOUR HEALTH BEFORE PREGNANCY

2.1. Did you have any illnesses or ailments before becoming pregnant?

- Yes
  - ☐ Diabetes
  - ☐ Heart/vascular disorder (including hypertension)
  - ☐ Autoimmune illness (rheumatoid illness, metabolic disorder, transplantation)
  - ☐ Systemic Lupus Erythematosus (SLE)
  - ☐ Anaemia (iron deficiency and thalassemia)
  - ☐ Kidney disease
  - ☐ Treated tuberculosis
  - ☐ HIV, hepatitis
  - ☐ Overweight
  - ☐ Neurological illness (such as epilepsy)
  - ☐ Lung illness (such as asthma)
  - ☐ Mental disorder (such as depression)
  - ☐ Other: i)________________ii)_________________
- No

2.2 a) How much did you weigh before pregnancy?

2.2 b) What is your height?

2.3 How would you assess your health for the time being. How would you describe your health?

... good / neither good nor bad / poor?

2.4 Describe your situation: Not troubled, A little troubled, Very troubled or Extremely troubled ...

a)... being constantly afraid or anxious?

- not troubled / a little troubled /very troubled / extremely troubled

b)... a sense of hopelessness for the future?

- not troubled / a little troubled /very troubled / extremely troubled

c)... a sense of loneliness?

- not troubled / a little troubled /very troubled / extremely troubled

---

We have some questions about how you planned this pregnancy and if you used birth control/contraception.

2.5 Was this pregnancy planned?

- Yes (go to part 3)
- No
- Do not know/unsure

2.5 If the pregnancy was not planned or you are not sure if it was planned, did you use any form of contraception to avoid pregnancy?

Yes → 2.7 | No → 2.8

2.7 What did you use?

- Barrier methods (condom, diaphragm)
- Non-hormonal methods/natural methods (interrupted intercourse/safe periods, breast feeding)
- Hormonal contraceptives (The pill, mini-pill, pregnancy prevention patches, vaginal ring)
- LARC (hormonal and/or copper spiral/IUD, contraceptive injection)
- Other (specify) ______________

2.8 Why did you not want to use birth control?

- ☐ Too expensive
- ☐ Did not have enough information about different methods/options
- ☐ I did not know where I could get hold of these
- ☐ No access to doctor/nurse
- ☐ Side effects
- ☐ Religious reasons
- ☐ Husband/partner/family did not want it
- ☐ Other (specify)__________________

3. OBSTETRIC CLINICAL HISTORY

3.1 How many children have you born, in total (including your new child)?

3.2 How many births have you had (past week 23)?

3.3 How many of your children were born in Norway (including your newborn)?

3.4 Have you had difficulties in previous pregnancies and births?

- Yes, which:
  - ☐ Cesarean section
  - ☐ Nausea during pregnancy
  - ☐ Hemorrhages/bleeding/anaemia
  - ☐ High blood pressure
  - ☐ Preeclampsia
  - ☐ Deep vein thrombosis (blood clot in the leg)
  - ☐ Gestational diabetes
  - ☐ Low-lying placenta
  - ☐ Abruptio placenta
  - ☐ Urinary tract infection
  - ☐ Symphysiolysis
  - ☐ Premature birth (<37 weeks)
  - ☐ Premature birth (<34 weeks)
  - ☐ Early rupture of membrane
  - ☐ Intrauterine growth retardation (decreasing growth indicated by series measurements)
  - ☐ Foetal death
  - ☐ Congenital abnormalities in foetus
  - ☐ Sphincter rupture (grade 3+4)
  - ☐ Postpartum depression
  - ☐ Other (please specify):_________________
- No, first birth
- No, I have not had any complications

4. CURRENT PREGNANCY

4.1 Were you pregnant with your newborn child when you came to Norway?

- Yes
- No
- Do not remember/do not know

4.2 Did you receive any form of health care for the pregnancy before birth from a health care provider (doctor, nurse, midwife) in Norway?

- Yes
- No

4.3 Who provided health care for your pregnancy in Norway?

- ☐ GP/Family doctor
- ☐ Specialist (obstetrician) at the hospital
- ☐ The midwife at the health clinic
- ☐ Other __________

4.4 How many weeks pregnant were you when you first received health care for this pregnancy in Norway?

4.5 Did you experience any difficulties in this pregnancy?

- Yes, which
  - ☐ Cesarean section
  - ☐ Nausea during pregnancy
  - ☐ Anaemia
  - ☐ High blood pressure
  - ☐ Preeclampsia
  - ☐ Deep vein thrombosis (blood clot in the leg)
  - ☐ Gestational diabetes
  - ☐ Low-lying placenta
  - ☐ Abruptio placenta
  - ☐ Urinary tract infection

- Symphysiolysis
- Premature birth (<37 weeks)
- Premature birth (<34 weeks)
- Early rupture of membrane
- Intrauterine growth retardation (decreasing growth indicated by series measurements)
- Foetal death
- Congenital abnormalities in foetus
- Postpartum depression
- Other (please specify):________________
- No, I had no complications

4.6. Which of the following offers did you accept during pregnancy?

- Municipal help (pregnancy course, prepare for birthing course, parental guidance)
- Other offers from non-governmental organizations (Bydelsmødre etc.)
- Contact with health care providers in your home country
- Alternative medicine/rituals
- Child Welfare Services
- Ultrasound foster diagnostics at the hospital (for special patient groups)
- Routine ultrasound, Week 18
- Other (please specify)_______________

4.7. Of the offers mentioned above, are there any you would have liked to use but felt they were not available during your pregnancy?

- Yes, specify (from the options above)_________________
- No

4.8. Have the following factors prevented you from taking advantage of an offer from the public health service?

- Practical limitations (transportation, work, lack of time) Yes – No
- Language barriers Yes – No
- Lack of information about offers (not aware they existed, did not know how Norway's health care system works, did not think I was entitled) Yes – No
- Afraid that it could affect my visa/residency application process Yes – No
- Afraid of medical examinations and tests Yes – No
- Other (please specify): ________________

4.9. What were your <u>2 main sources</u> of information about pregnancy and birth during this pregnancy?

- Previous pregnancies/births
- Family/friends
- Religious/spiritual leader
- Health care providers
- Offers from my neighbourhood/district (courses)
- Mass media (books, TV, internet)
- Other (please specify): ________________

4.10. Did you get <u>enough</u> information about the following topics in the course of this pregnancy/birth?

- Physical changes during pregnancy Yes – No
- Emotional changes (feelings) during pregnancy Yes – No
- Recommended medical tests (HIV, hepatitis) Yes – No
- Nutrition during pregnancy Yes – No
- Signs that the birth had started Yes – No
- The various phases of birth Yes – No
- Pain relief during childbirth Yes – No
- Changes in mood after the birth Yes – No
- Breastfeeding Yes – No
- Infant formula Yes – No
- Where and who you could contact if you needed advice or had questions about your health or your newborn child's health Yes – No
- Family planning and birth control Yes – No

4.11. Did you take daily vitamin supplements during pregnancy?

- Yes (skip to question 4.12
- No (go to next question)

4.12. If NO, why not?

- ☐ Did not know why it should be taken
- ☐ Could not find it at the store
- ☐ Too expensive
- ☐ Did not need it
- ☐ Was not told/asked about taking it
- ☐ Other (please specify):____________

4.13. Which of the following statements best describes your habits during pregnancy?

- Smoking: I did not smoke, I smoked occasionally, I smoked daily
- Snuff: I did not take snuff, I took snuff occasionally, I took snuff daily
- Alcohol: I did not drink alcohol, I drank alcohol occasionally, I drank alcohol every day

5. BIRTH

5.1. How many weeks were you pregnant before giving birth?

5.2. How many baby(is) were born?

5.3. Were any of the following procedures performed during the birth?

- ☐ Labour induction
- ☐ Use of a vacuum
- ☐ Use of forceps
- ☐ Cesarean section
- ☐ Episiotomy (cutting near the opening of the vagina)
- ☐ Epidural/Spinal anaesthesia as pain relief
- ☐ Pudendal blockade as pain relief
- ☐ Other (please specify):__________________

5.4. Did you have any complications during the birth?

- Yes
  - ☐ Sphincter rupture (grade 3-4)
  - ☐ Bleeding that needed transfusion
  - ☐ The infant was moved to the neonatal ward
  - ☐ Use of antibiotics
  - ☐ Other (please specify):__________________
- No

5.5. If your child was born via caesarean section, what was the reason for it?

- It was scheduled because the doctor recommended it for medical reasons
- It was planned, but you do not know why
- It was scheduled because you wanted it, but not for medical reasons
- It was not planned, but the birth took a long time
- It was not planned but the baby/foetus was in danger
- It was not planned but you were in danger
- It was not planned and you do not know why it was done
- Other (please specify):_______________

5.6. Are you satisfied with the help you received from the health care provider to relieve your pain?

- Yes
- No
- Not a vaginal birth, I had a caesarean section

5.7. Were you allowed to have a family member or other support person (including a doula) with you in the birthing room?

- Yes
- No

5.8. Do you feel that the duration of your hospitalisation after birth was:

- Too short
- OK/suitable
- Too long

6. OVERALL EXPERIENCE OF PREGNANCY CARE RECEIVED

6.1. Did the health care provider refuse any care, special practice or ritual during or after birth that you requested?

Yes

No (go to question 6.4)

6.2. If yes, what were these wishes?

i)____________ ii)___________

6.3. If YES, what reason did the health care provider give for not allowing your wishes?

i)____________ ii)___________

6.4. Is there anything you think the health care provider could have done differently or better during the pregnancy, birth or after birth?

- Yes, please specify what could have been done differently or better

---

and by whom____

- No

6.5. Overall, were you satisfied with the health care you got? Did you feel welcome, was the health care provider helpful and respectful?

a) During pregnancy – Always – Sometimes – Rarely – Never

b) During the birth – Always – Sometimes – Rarely – Never

c) After birth – Always – Sometimes – Rarely – Never

6.6. Did you understand the information the health care provider tried to convey to you?

a) During pregnancy – Always – Sometimes – Rarely – Never

b) During the birth – Always – Sometimes – Rarely – Never

c) After birth – Always – Sometimes – Rarely – Never

6.7. Do you think you would have understood the information that was conveyed to you better in another language, such as your native language?

- Yes
- No

6.8. Were you offered an interpreter?

a) During pregnancy – yes/no/did not need an interpreter

b) During the birth – yes/no/did not need an interpreter

c) After birth – yes/no/did not need an interpreter

6.9. If you had someone there to interpret for you, who was it?

- Partner/other adult family member/friend
- Child (<18 years)
- Health care provider
- Professional interpreter
- Other_______

6.10. Were you happy with their interpretation?

- Yes
- No

6.11. The health care provider asked me if I had any questions.

Always – Sometimes – Rarely – Never

6.12. I felt that my concerns were taken seriously by the health care providers

Always – Sometimes – Rarely – Never

6.13. I had to wait a long time before I got help.

a) During pregnancy – Always – Sometimes – Rarely – Never

b) During the birth – Always – Sometimes – Rarely – Never

c) After birth – Always – Sometimes – Rarely – Never

6.14. The health care providers made decisions without asking my opinion

a) During pregnancy – Always – Sometimes – Rarely – Never
b) During the birth – Always – Sometimes – Rarely – Never
c) After birth – Always – Sometimes – Rarely – Never

6.15. The health care provider spent enough time explaining things to me.

a) During pregnancy – Always – Sometimes – Rarely – Never
b) During the birth – Always – Sometimes – Rarely – Never
c) After birth – Always – Sometimes – Rarely – Never

6.16. Overall, do you feel that you were treated differently by the health care providers, compared with other people? (i.e. because of language, culture, religion)?

Always – Sometimes – Rarely – Never

6.17. If yes, why do you think you were treated differently?

- ☐ Language
- ☐ Culture
- ☐ Ethnic background
- ☐ Skin colour
- ☐ Religion
- ☐ Migration status/immigrant background
- ☐ Other reasons (please specify):________________

7. MIGRATION

7.1. What was the legal basis for your residency permit in Norway? Is it ...

- Work/partner's work
- Reunion with family
- Marriage
- Refuge (resettlement refugee, quota refugee, humanitarian grounds, asylum)
- Education
- Undocumented
- Other (please specify):_______________

7.2. Did you live at a reception centre for asylum-seekers while you were pregnant with this child?

- Yes
- No

7.3. If yes, how long did you live there?

7.4. Do you have a work permit in Norway?

- Yes
- No

7.5 How satisfied or dissatisfied are you with your life after coming to Norway?

- Dissatisfied
- Neither satisfied or dissatisfied
- Satisfied

7.6 How satisfied or dissatisfied were you with life in your home country before you came to Norway?

- Dissatisfied
- Neither satisfied or dissatisfied
- Satisfied
