# Supplementary material for: Newly Arrived Migrant Women’s Experience of Maternity Health Information: A Face-to-Face Questionnaire Study in Norway
Source: Int J Environ Res Public Health. 2021 Jul 15;18(14):7523. doi: 10.3390/ijerph18147523 (PMC8307311; doi:10.3390/ijerph18147523)
Supplement: Supplementary file 1 [file ijerph-18-07523-s001.zip › Table S1.pdf]

Offered professional interpreter during pregnancy	Needed and got	Needed but did not get	Did not need
Women´s region of birth (GBD)			
Central/Eastern Europe and Central Asia	27 (20.5)	22 (16.7)	83 (62.9)
Latin America and Caribbean	2 (15.4)	3 (23.1)	8 (61.5)
North Africa and Middle East	28 (36.8)	11 (14.5)	37 (48.7)
South Asia	10 (12.3)	13 (16.0)	58 (71.6)
Southeast Asia, East Asia and Oceania	3 (8.1)	10 (27.0)	24 (64.9)
Sub-Saharan Africa	25 (40.3)	15(24.2)	22 (35.5)
Majority language proficiency			
Low	41 (36.6)	22 (19.6)	49 (43.8)
Moderate	45 (26.0)	38 (22.0)	90 (52.0)
High	9 (7.8)	14 (12.1)	93 (80.2)
Reason for migration			
Refugee	17 (41.5)	11 (26.8)	13 (31.7)
Family reunification	53 (29.0)	33 (18.0)	97 (53.0)
Education/work	25 (14.1)	30 (16.9)	122 (68.9)
Financial level			
Low	26 (29.5)	28 (31.8)	34 (38.6)
High	69 (22.0)	46 (14.7)	198 (63.3)
Education			
No completed school	8 (50.0)	6 (37.5)	2 (12.5)
Primary/secondary school	64 (42.4)	29 (19.2)	58 (38.4)
University	23 (9.8)	39 (16.7)	172 (73.5)
Employment			
Unemployed	67 (38.7)	34 (19.7)	72 (41.6)
Employed	28 (12.3)	40 (17.5)	160 (70.2)

Table S1: Characteristics of all study participants according to if the women needed and got an interpreter; needed but did not get an interpreter and those who did not need a professional interpreter, n (%)
